# Long-term trends of inequalities in mortality in 6 European countries

**DOI:** 10.1007/s00038-016-0922-9

**Published:** 2016-12-09

**Authors:** Rianne de Gelder, Gwenn Menvielle, Giuseppe Costa, Katalin Kovács, Pekka Martikainen, Bjørn Heine Strand, Johan P. Mackenbach

**Affiliations:** 1 Department of Public Health, Erasmus MC, Rotterdam, The Netherlands; 2 UPMC Univ Paris 06, INSERM, Institut Pierre Louis d’Epidémiologie et de Santé Publique (IPLESP UMRS 1136), Sorbonne Universités, Paris, France; 3 Department of Clinical Medicine and Biology, University of Turin, Turin, Italy; 4 Demographic Research Institute, Budapest, Hungary; 5 Department of Sociology, University of Helsinki, Helsinki, Finland; 6 Division of Epidemiology, Norwegian Institute of Public Health, Oslo, Norway

**Keywords:** Mortality, Socioeconomic inequalities, Trends, Europe

## Abstract

**Objectives:**

We aimed to assess whether trends in inequalities in mortality during the period 1970–2010 differed between Finland, Norway, England and Wales, France, Italy (Turin) and Hungary.

**Methods:**

Total and cause-specific mortality data by educational level and, if available, occupational class were collected and harmonized. Both relative and absolute measures of inequality in mortality were calculated.

**Results:**

In all countries except Hungary, all-cause mortality declined strongly over time in all socioeconomic groups. Relative inequalities in all-cause mortality generally increased, but more so in Hungary and Norway than elsewhere. Absolute inequalities often narrowed, but went up in Hungary and Norway. As a result of these trends, Hungary (where inequalities in mortality where almost absent in the 1970s) and Norway (where inequalities in the 1970s were among the smallest of the six countries in this study) now have larger inequalities in mortality than the other four countries.

**Conclusions:**

While some countries have experienced dramatic setbacks, others have made substantial progress in reducing inequalities in mortality.

**Electronic supplementary material:**

The online version of this article (doi:10.1007/s00038-016-0922-9) contains supplementary material, which is available to authorized users.

## Introduction

Widening relative and/or absolute inequalities in mortality over the past two decades have been reported from many countries (Borrell et al. 2008; Fawcett et al. 2005; Jemal et al. 2008; Krieger et al. 2008; Mackenbach et al. 2003; Mackenbach et al. 2014; Martikainen et al. 2014; Tarkiainen et al. 2012; Strand et al. 2010, 2014), but studies of long-term trends stretching over three or more decades are rare, and are usually limited to a single country. Examples include studies of long-term trends in the United States (Krieger et al. 2008), Finland (Martikainen et al. 2014), Norway (Strand et al. 2014), France (Menvielle et al. 2007), England and Wales (Capewell and Graham 2010) and Italy (Stringhini et al. 2015).

While short-term trends are important for monitoring, for example because they reflect the effect of changes in exposure to determinants of mortality with a relatively immediate impact, such as improvements in medical treatment or road safety, long-term trends may provide insights into how secular changes in mortality and its determinants play out in the evolving pattern of health inequalities. For example, high-income countries are in an advanced stage of the epidemiologic transition (Olshansky and Ault 1986; Omran 1971), with rapidly but differentially declining rates of cardiovascular disease mortality and widening inequalities in cardiovascular disease mortality as a result (Avendano et al. 2006; Kunst et al. 1999; Marmot and McDowall 1986). Such secular changes can only be captured on a time-scale of three or four decades.

Previous studies have provided a mixed picture of long-term trends in inequalities in mortality. Relative inequalities (e.g., inequalities expressed in terms of a rate ratio, indicating the strength of the association between socioeconomic position and mortality regardless of the absolute level of mortality) seem to have universally widened, even in the highly developed welfare states of Western Europe, but for absolute inequalities (e.g., inequalities expressed in terms of a rate difference, indicating the absolute mortality excess in lower as compared to higher socioeconomic groups) both widening and narrowing have been reported (Martikainen et al. 2014; Strand et al. 2014; Shkolnikov et al. 2012; Stringhini et al. 2015; Menvielle et al. 2007). Because almost no studies have quantitatively compared these trends between countries, it is unknown whether countries differ in the timing of widening or narrowing of inequalities in mortality.

We therefore analyzed trends in socioeconomic inequalities in mortality over four decades, using a unique dataset with harmonized data from six European countries. In addition to the Western European countries mentioned above, this dataset also covers Hungary, thereby providing a first analysis of long-term trends in inequalities in mortality in Central/Eastern Europe—a part of the subcontinent whose political history had a profound impact on mortality and life expectancy (Mackenbach 2013).

## Methods

### Data

In this study mortality data were used from Finland, Norway, England and Wales, France, Italy and Hungary, based on a total of 269,550,158 person years, covering the period 1970–2010. Key characteristics of the data are shown in Table 1. Data were harmonized to enhance between- and within-country comparability and contained information on sex, age, educational level, occupational class, and cause-specific mortality. The educational distribution of each country’s population is presented in web appendix Table 1. Most data sets covered the entire national territories, but in Italy data from the Turin region only were available. Previous studies have shown that patterns and trends observed in this regional population correspond well to those seen at the national level (Federico et al. 2013; Marinacci et al. 2013).

**Table 1:** Key characteristics of the datasets used in the analysis

Country	Type	Years	Census date	Geographic coverage	Ages included	Number of deaths	Number of person years
Finland	Longitudinal	Dec 31, 1970 to Dec 31, 1980	31.12.1970	National	35–79	310,253	19,401,076
		Dec 31, 1980 to Dec 31, 1990	31.12.1980			297,925	22,132,564
		Dec 31, 1990 to Dec 31, 2000	31.12.1990			271,246	25,148,551
		Dec 31, 2000 to Dec 31, 2010	31.12.2000			247,441	27,446,463
Norway	Longitudinal	Nov, 1970 to Dec, 1980	Nov, 1970	National	40–79	237,790	14,097,453
		Nov, 1980 to Dec, 1990	Nov, 1980			235,645	13,804,407
		Nov, 1990 to Dec, 2001	Nov, 1990			227,996	16,372,733
		Nov, 2001 to Dec, 2009	Nov, 2001			133,675	14,050,440
England/Wales	Longitudinal	Apr 25, 1971 to Apr 4,1981	25.4. 1971	National	35–79	40,543	2,315,725
		Apr 5, 1981 to Apr 20,1991	5.4.1981			36,715	2,453,660
		Apr 21, 1991 to Apr 28, 2001	21.4.1991			31,472	2,579,226
		Apr 29, 2001 to Dec 31, 2009	29.4. 2001			22,009	2,431,057
France	Longitudinal	10.1.1975 to 9.1.1982	10.1.1975	National	35–79	16,940	1,397,501
		10.1.1982 to 9.1.1990	10.1.1982			19,236	1,689,497
		10.1.1990 to 9.1.1999	10.1.1990			19,331	2,113,937
		10.1.1999 to 31.12.2007	10.1.1999			15,663	1,745,199
Italy (Turin)	Longitudinal	Oct 24, 1971 to Oct 24, 1981	24.10.1971	City	35–79	58,426	4,689,534
		Oct 25, 1981 to Oct 19, 1991	25.10.1981			56,947	5,118,272
		Oct 20, 1991 to Oct 20, 2001	20.10.1991			46,181	4,736,315
		Oct 21, 2001 to Dec 31, 2010	21.10.2001			35,056	4,283,032
Hungary	CS, unlinked	1971–1974	1.1.1973	National	35–79	340,511	19,754,780
		1978–1981	1.1.1980			393,590	20,180,700
		1988–1991	1990			385,974	20,576,688
		1999–2002	2001			369,773	21,031,348

Data from England and Wales and France concern a 1% representative sample of the total population

In France, only those born in mainland France were included (excluding overseas territories and abroad)

*CS* cross-sectional

Data from Finland, Norway, England and Wales, France and Italy (Turin) were generated in a longitudinal mortality follow-up after a census, in which mortality data were linked to socioeconomic information that had been recorded in the census; each decade of follow-up was divided in approximate 5-year periods for the analysis to allow a more fine-grained analysis of time-trends. Hungary has so-called cross-sectional unlinked data in which socioeconomic information on the population-at-risk comes from the census, and on the deceased comes from the death certificate.

We used two indicators of socioeconomic position: level of education and occupational class. Education was classified according to the International Standard Classification of Education 1997 (UNESCO 2006). Three groups were distinguished: ‘primary education and lower secondary education (ISCED 0, 1 and 2; ‘low’)’, ‘upper secondary education and post-secondary, non-tertiary education (ISCED 3 and 4; ‘middle’)’, and tertiary education (ISCED 5 and 6; ‘high’). In the datasets for 1981–1991 and 1991–2001 in England and Wales only two levels of education could be distinguished (‘low and middle’ vs ‘high’), and we therefore focussed on the periods 1971–1981 and 2001–2009 for the main analysis. Occupational class was classified following the Erikson–Goldthorpe–Portocarero (EGP) social class scheme (Erikson and Goldthorpe 1992). Four categories were distinguished: non-manual workers, manual workers, farmers and self-employed, but because the relative position of farmers and self-employed workers in the social hierarchy is ambiguous, and information on occupational class is less reliable for women, we only report inequalities between men in manual and non-manual occupations. Because data on education were available for all countries, whereas data on occupation were available for men in 4 countries only, inequalities by educational group are presented as main outcome.

Deaths were classified according to the 8th, 9th or 10th revision of the International Classification of Diseases (ICD). ICD-codes were harmonized following the scheme presented in web appendix Table 2. Our main outcome variables were all-cause mortality, and mortality due to cardiovascular disease (ICD-10 I00-I99), cancer (ICD-10 C00-D48), all other diseases (ICD-10 A00-B99, D5-H95 and J00-U85), and external causes (i.e., injuries; V01-Y98).

### Analysis

Age-standardized mortality rates by educational level, sex, country and time-period were calculated using the European Standard Population (Ahmad et al. 2001). Analyses were restricted to persons aged 35–79 for analyses by educational level (40–79 in Norway), and to persons aged 35–64 (active working population) for analyses by occupational class. Ages refer to age at death.

Educational inequalities in mortality were assessed with both relative and absolute measures, using the Relative Index of Inequality (RII) and the Slope Index of Inequality (SII) (Mackenbach and Kunst 1997; Mackenbach et al. 2008). The RII and SII are regression-based measures which take into account the distribution of education in a population, and adjust the relative position of each group to its share in the population, which increases comparability over time and between countries if there are substantial changes or differences in distribution of the population over socioeconomic groups. RIIs were calculated with Poisson regression with educational ‘rank’ as an independent variable, controlling for age (in 5-year age groups). Educational ‘rank’ was calculated for each education group (by country, sex and period) as the mean proportion of the population having a higher level of education. This ensures that all education groups (not only the lowest and highest) are taken into account, and that the magnitude of inequalities in mortality can be compared between countries even if their educational distributions are different. The RII is a relative measure which can be interpreted as the rate ratio of mortality among those with the very lowest educational level compared to those with the very highest educational level. SIIs were calculated from the RIIs and the age-standardized mortality rates (ASMR) in the general population using the formula: SII = 2 × ASMR × (RII − 1)/(RII + 1). The SII is an absolute measure which can be interpreted as the rate difference of mortality between those with the very lowest and those with the very highest educational level (Mackenbach and Kunst 1997; Mackenbach et al. 2008). We calculated 95% CIs using bootstrapping of 1000 replicas. As our analysis of mortality by occupational class only involved two classes (manual and non-manual), we used simple Rate Ratios and Rate Differences with the non-manual class as the reference group for this socioeconomic indicator.

## Results

In this study, a total of 3,850,338 deaths were observed. Tables 2 and 3 present all-cause mortality rates by education for each country and time-period, and changes in mortality rates over time. Full details on mortality rates by cause of death, sex, time-period and education in each country are given in web appendix Table 3. In the early 1970s, all-cause mortality was already highest among the lowest educated in all countries among both men and women, with the exception of Hungarian women for whom mortality was slightly higher among the high educated, and even 1.4 times higher among the higher educated in the early 1980s. Since the early 1980s, mortality gradually decreased over time in all educational groups, except in Hungary where mortality among the low and middle educated increased until the early 1980s (women) and early 1990s (men) and then also started to decline.

**Table 2:** Age-standardized all-cause mortality rates per 100.000 person years, by educational group, men and women

Country/period	Educational level							
	Total		Low		Middle		High	
	ASMR	95% CI	ASMR	95% CI	ASMR	95% CI	ASMR	95% CI
Finland								
1970–1974	2205.3	(2191.6–2219.9)	2307.7	(2291.6–2324.1)	1769.5	(1720.6–1815.8)	1669.2	(1621.7–1716.2)
1975–1979	2014.2	(2000.9–2028.2)	2128.9	(2113.8–2143.8)	1598.9	(1556.1–1638.1)	1498.2	(1461.5–1536.6)
1980–1984	1812.5	(1800.7–1823.5)	1934.9	(1921.6–1949.2)	1575.4	(1537.1–1611.1)	1290.6	(1260.4–1324.3)
1985–1989	1673.1	(1660.9–1683.3)	1820.2	(1806.8–1834.2)	1485.1	(1453.9–1515.6)	1156.7	(1131.8–1183.7)
1990–1994	1485.2	(1475.3–1495.8)	1655.3	(1642.5–1668.8)	1344.4	(1318.4–1371.7)	997.7	(975.5–1020.9)
1995–1999	1304.9	(1296.2–1314.6)	1499.0	(1484.3–1511.6)	1213.6	(1192.4–1236.7)	842.8	(824.2–861.0)
2000–2004	1130.6	(1123.1–1139.2)	1363.2	(1349.7–1375.9)	1063.8	(1047.1–1080.6)	718.3	(703.8–733.1)
2005–2009	1016.6	(1009.8–1024.2)	1294.8	(1280.2–1309.4)	1003.5	(990.4–1018.2)	627.6	(615.8–639.0)
Percent change last—first (95% CI)	−53.9%	(−54.3 to −53.5%)	−43.9%	(−44.6 to −43.2%)	−43.3%	(−44.9 to −41.6%)	−62.4%	(−63.6 to −61.1%)
Absolute change last—first (95% CI)	−1188.7	(−1204.6 to −1172.8)	−1012.9	(−1034.7 to −991.1)	−766.0	(−815.6 to −716.4)	−1041.6	(−1090.3 to −992.9)
Norway								
1970–1974	1727.1	(1716.0–1738.0)	1827.9	(1810.0–1845.5)	1595.6	(1578.1–1615.6)	1329.7	(1287.5–1372.2)
1975–1979	1711.9	(1700.8–1723.6)	1838.6	(1818.5–1860.4)	1599.6	(1574.9–1616.0)	1255.0	(1216.2–1293.0)
1980–1984	1628.1	(1617.6–1638.8)	1780.5	(1764.6–1801.0)	1514.2	(1494.6–1530.2)	1180.7	(1149.7–1211.5)
1985–1989	1594.0	(1581.9–1603.9)	1792.1	(1768.9–1812.8)	1470.3	(1449.8–1487.7)	1085.0	(1053.5–1115.3)
1990–1994	1412.3	(1402.0–1422.0)	1656.4	(1638.5–1675.8)	1310.6	(1293.4–1323.4)	945.7	(917.0–967.1)
1995–1999	1245.4	(1236.6–1254.7)	1572.3	(1554.2–1596.1)	1146.9	(1133.5–1162.7)	800.8	(782.5–818.8)
2000–2004	1028.2	(1020.2–1035.3)	1381.2	(1361.1–1405.7)	953.4	(940.5–965.9)	660.3	(642.4–677.8)
2005–2009	933.8	(922.9–943.8)	1284.2	(1253.5–1310.3)	892.4	(880.0–906.2)	596.5	(578.5–614.0)
Percent change last—first (95% CI)	−45.9%	(−46.7 to −45.2%)	−29.7%	(−31.3 to −28.2%)	−11.1%	(−45.4 to −42.8%)	−55.1%	(−57.3 to 52.9%)
Absolute change last—first (95% CI)	−793.3	(−810.1 to −776.5)	−543.7	(−574.1 to −513.3)	−703.2	(−732.0 to −674.4)	−733.2	(−781.9 to −684.5)
England and Wales								
1970–1974	1969.3	(1935.0–2002.1)	1965.1	(1928.3–2005.4)	NA	NA	1493.0	(1333.2–1655.0)
1975–1979	1868.1	(1836.0–1902.0)	1898.5	(1860.6–1935.9)	NA	NA	1220.7	(1106.9–1332.8)
1980–1984	1650.6	(1622.5–1682.0)	1703.0	(1665.3–1735.6)	NA	NA	1063.8	(971.5–1154.9)
1985–1989	1486.7	(1456.2–1516.7)	1544.9	(1515.3–1576.5)	NA	NA	976.2	(895.2–1054.0)
1990–1994	1334.1	(1305.5–1360.3)	1389.4	(1357.3–1421.4)	NA	NA	895.6	(833.2–959.7)
1995–1999	1150.0	(1125.6–1172.6)	1215.1	(1184.9–1245.8)	NA	NA	755.0	(708.6– 806.1)
2000–2004	994.1	(968.9–1014.2)	1070.4	(1043.1–1097.9)	NA	NA	677.0	(629.4–722.6)
2005–2009	809.9	(785.5–833.1)	876.7	(846.6–906.7)	NA	NA	559.2	(514.6–606.8)
Percent change last—first (95% CI)	−58.9%	(−60.3 to −57.5%)	−55.4%	(−57.1 to −53.8%)	NA	NA	−62.5%	(−67.3 to −57.2%)
Absolute change last—first (95% CI)	−1159.4	(−1200.5 to −1118.3)	−1088.4	(−1137.3 to −1039.5)	NA	NA	−933.8	(−1101.2 to −766.4)
France								
1975–1979	1461.9	(1428.5–1495.3)	1565.1	(1525.7–1601.8)	1155.1	(1061.9–1261.0)	861.9	(749.5–982.4)
1980–1984	1466.4	(1424.1–1508.8)	1588.4	(1536.3–1642.8)	1171.7	(1067.8–1279.0)	903.9	(769.4–1046.6)
1985–1989	1423.7	(1386.9–1460.4)	1554.5	(1508.2–1602.6)	1181.9	(1095.9–1269.2)	880.0	(772.1–989.1)
1990–1994	1199.5	(1171.1–1227.9)	1351.7	(1315.6–1391.5)	1030.8	(972.2–1084.8)	674.4	(599.1–749.5)
1995–1999	1160.8	(1130.4–1191.1)	1356.5	(1312.8–1404.1)	986.7	(934.8–1404.1)	630.0	(556.7–706.8)
2000–2004	996.6	(971.9–1021.4)	1196.6	(1155.8–1235.8)	899.8	(854.3–940.2)	548.5	(491.8–609.8)
2005–2009	933.4	(906.9–959.9)	1150.3	(1101.8–1196.7)	865.3	(822.1–907.5)	576.5	(520.8–633.2)
Percent change last—first (95% CI)	−36.2%	(−38.5 to −33.8%)	−26.5%	(−30.1 to−23.1%)	−25.1%	(−31.7 to −18.0%)	−33.1%	(−43.2 to −19.9%)
Absolute change last—first (95% CI)	−528.5	(−571.1 to −485.9)	−414.8	(−475.6 to −354.0)	−289.8	(−398.1 to −181.5)	−285.4	(−414.7 to −156.1)
Italy (Turin)								
1970–1974	1694.5	(1669.0–1718.6)	1729.6	(1703.2–1758.0)	1591.0	(1501.0- 1678.2)	1321.8	(1229.2–1414.7)
1975–1979	1607.5	(1584.1–1632.2)	1649.9	(1624.6–1676.9)	1437.0	(1351.8–1513.5)	1289.8	(1200.9–1385.5)
1980–1984	1429.4	(1407.2–1451.0)	1482.2	(1458.1–1505.6)	1259.1	(1196.3–1328.9)	1034.9	(962.5–1098.7)
1985–1989	1251.0	(1232.2–1270.6)	1309.9	(1286.9–1332.7)	1096.0	(1039.3–1148.5)	910.9	(848.5–975.7)
1990–1994	1120.3	(1102.2–1138.3)	1192.7	(1171.7–1214.4)	978.6	(933.2–1025.6)	808.9	(758.4–860.3)
1995–1999	941.2	(924.8–957.3)	1019.9	(999.0–1041.9)	792.0	(754.0–830.5)	669.1	(623.3–712.9)
2000–2004	807.5	(793.4–821.6)	904.5	(884.3–924.6)	668.6	(638.5–698.7)	571.8	(533.8–609.1)
2005–2009	715.2	(699.7–731.6)	826.1	(803.8–850.0)	596.4	(565.0–625.6)	485.7	(448.9–524.4)
Percent change last—first (95% CI)	−57.8%	(−59.0 to −56.6%)	−52.2%	(−53.7 to −50.7%)	−62.5%	(−65.1 to −59.6%)	−63.3%	(−66.9 to −59.1%)
Absolute change last—first (95% CI)	−979.3	(−1008.8 to −949.8)	−903.5	(−939.3 to −867.7)	−994.6	(−1088.2 to −901.0)	−836.1	(−936.2 to −736.0)
Hungary								
1970–1974	1997.4	(1989.3–2004.7)	2033.4	(2023.9–2044.4)	1748.1	(1714.1–1782.5)	1769.3	(1730.3–1810.9)
1980–1984	2296.8	(2287.8–2305.2)	2373.6	(2363.9–2386.3)	1970.4	(1944.4–2005.3)	1998.4	(1966.1–2029.9)
1990–1994	2436.0	(2426.5–2445.2)	2650.9	(2640.2–2662.6)	2103.9	(2074.4–2134.5)	1409.4	(1385.2–1433.8)
2000–2004	2194.5	(2183.9–2202.3)	2623.7	(2614.2–2638.5)	1469.5	(1449.4–1484.2)	1020.4	(1003.3–1033.3)
Percent change last—first (95% CI)	+9.9%	(9.2–10.5%)	+29.0%	(28.2–29.9%)	−15.9%	(−17.8 to −13.9%)	−42.3%	(−43.9 to −40.8%)
Absolute change last—first (95% CI)	+197.1	(185.1–209.1)	+590.4	(574.5–606.2)	−278.6	(−316.9 to −240.2)	−749.0	(−792.0 to −706.0)

In England and Wales, mortality rates among ‘low’ and ‘middle’ educated were combined because ‘middle’ education is not available in the 1980s and 1990s

*ASMR* age-standardized mortality rate, *NA* not available, *CI* confidence interval

**Table 3:** Age-standardized all-cause mortality rates per 100.000 person years, by educational group, women

Country/period	Educational level							
	Total		Low		Middle		High	
	ASMR	95% CI	ASMR	95% CI	ASMR	95% CI	ASMR	95% CI
Finland								
1970–1974	1041.7	(1033.8–1049.3)	1076.9	(1068.4–1085.2)	809.7	(781.6–836.7)	794.5	(763.0–824.6)
1975–1979	881.8	(874.6–888.7)	916.2	(907.4–923.9)	714.1	(691.8–736.0)	673.3	(647.3–700.0)
1980–1984	794.8	(788.4–801.2)	830.1	(822.7–837.2)	674.4	(655.3–691.8)	591.9	(570.7–613.4)
1985–1989	758.2	(751.8–764.3)	800.0	(792.0–807.6)	666.8	(651.3–682.6)	568.5	(549.6–587.7)
1990–1994	679.2	(673.3–684.7)	739.8	(732.5–747.1)	584.2	(571.1–597.1)	508.4	(492.1–525.1)
1995–1999	595.3	(590.0–600.2)	669.8	(661.6–677.9)	524.4	(513.7–535.7)	434.3	(421.0–447.7)
2000–2004	527.9	(523.1–532.7)	641.3	(632.4–649.7)	470.0	(461.2–479.8)	374.3	(363.2–384.4)
2005–2009	469.7	(464.6–474.1)	613.1	(602.0–623.4)	437.3	(429.3–445.4)	336.1	(327.2–344.4)
Percent change last—first (95% CI)	−54.9%	(−55.4 to−54.3%)	−43.1%	(−44.2 to −42.0%)	−46.0%	(−48.2 to −43.9%)	−57.7%	(−59.6 to −55.7%)
Absolute change last—first (95% CI)	−572.0	(−581.1 to−562.9)	−463.8	(−477.4 to −450.2)	−372.4	(−401.1 to −343.7)	−458.4	(−490.4 to −426.4)
Norway								
1970–1974	938.3	(929.5–945.8)	1000.7	(992.4–1011.2)	774.5	(758.1–786.5)	689.2	(658.5–729.9)
1975–1979	874.5	(865.1–884.4)	935.8	(927.6–947.8)	753.4	(737.6–768.3)	653.6	(623.0–681.1)
1980–1984	799.3	(792.0–806.4)	857.5	(848.7–866.3)	700.2	(687.5–711.0)	597.1	(574.7–623.2)
1985–1989	802.3	(795.2–810.1)	872.8	(862.6–885.6)	700.5	(689.2–712.5)	586.4	(561.1–620.4)
1990–1994	737.5	(730.9–744.1)	835.6	(826.2–846.7)	645.8	(635.1–656.3)	507.5	(486.4–522.8)
1995–1999	693.0	(686.7–700.1)	849.3	(837.5–861.5)	602.5	(591.6–611.4)	447.5	(432.2–462.7)
2000–2004	612.8	(606.1–619.2)	789.8	(778.1–800.6)	537.9	(528.8–547.3)	403.6	(391.5–418.4)
2005–2009	572.1	(564.4–579.7)	764.3	(749.0–781.6)	513.5	(500.4–523.2)	372.3	(360.9–388.6)
Percent change last—first (95% CI)	−39.0%	(−40.0 to −38.1%)	−23.6%	(−25.3 to −21.6%)	−33.7%	(−35.7 to −31.6%)	–46.0%	(−49.6 to −42.1%)
Absolute change last—first (95% CI)	−366.2	(−377.9 to −354.5)	−236.4	(−257.4 to −215.4)	−261.0	(−280.8 to −241.2)	–316.9	(−357.4 to −276.4)
England and Wales								
1970–1974	1114.9	(1091.7–1138.8)	1090.8	(1066.0–1114.2)	NA	NA	828.5	(724.5–948.8)
1975–1979	1001.1	(979.8–1021.8)	1009.4	(986.0–1034.4)	NA	NA	686.2	(602.0–776.2)
1980–1984	929.9	(909.3–951.4)	942.6	(922.4–964.3)	NA	NA	677.8	(596.4–760.2)
1985–1989	842.7	(822.5–863.5)	857.0	(837.3–877.6)	NA	NA	630.9	(559.9–701.5)
1990–1994	795.6	(776.3–815.3)	810.9	(789.2–832.5)	NA	NA	557.2	(505.0–617.4)
1995–1999	721.2	(702.9–740.9)	747.0	(725.6–769.3)	NA	NA	459.2	(415.7–505.3)
2000–2004	639.7	(623.5–656.7)	673.9	(655.0–693.7)	NA	NA	443.4	(403.0–484.4)
2005–2009	569.7	(551.0–587.8)	610.8	(590.2–634.4)	NA	NA	417.5	(378.2–459.5)
Percent change last—first (95% CI)	−48.9%	(−50.8 to −46.7%)	−44.0%	(−46.4 to −41.6%)	NA	NA	−49.6%	(−57.0 to −39.7%)
Absolute change last—first (95% CI)	−545.2	(–575.1 to −515.3)	–480.0	(–512.7 to −447.3)	NA	NA	−411.0	(−530.3 to −291.7)
France								
1975–1979	665.0	(645.2–684.8)	681.0	(660.0–703.3)	561.3	(489.4–641.5)	485.8	(372.7–591.8)
1980–1984	619.8	(595.4–644.2)	637.4	(611.4–664.4)	523.3	(436.5–613.6)	480.9	(346.5–625.6)
1985–1989	589.9	(568.9–610.9)	614.0	(590.9–637.0)	478.3	(411.4–546.9)	366.1	(277.8–458.7)
1990–1994	510.2	(493.5–526.8)	544.9	(524.0–566.0)	412.0	(372.6–454.4)	337.3	(270.4–408.8)
1995–1999	489.9	(471.9–507.8)	548.2	(524.3–573.2)	364.6	(328.1–401.9)	330.3	(265.0–402.0)
2000–2004	424.1	(409.2–438.9)	475.4	(454.2–495.7)	379.1	(348.6–406.5)	287.2	(241.5–331.2)
2005–2009	427.9	(411.1–444.6)	496.7	(472.3–526.1)	381.5	(351.5–412.8)	282.6	(238.3–327.2)
Percent change last—first (95% CI)	−35.7%	(−38.8 to −32.4%)	−27.1%	(−31.5 to −22.7%)	−32.0%	(−41.8 to −20.4%)	−41.8%	(–55.5 to −20.4%)
Absolute change last—first (95% CI)	−237.1	(−263.0 to −211.2)	−184.3	(−218.8 to −149.8)	−179.8	(−261.8 to −97.8)	−203.2	(−321.4 to −85.0)
Italy (Turin)								
1970–1974	866.2	(851.2–881.5)	877.6	(862.0–893.5)	715.7	(654.7–776.4)	615.1	(499.0–741.1)
1975–1979	809.4	(794.2–824.3)	819.7	(804.5–835.0)	687.1	(634.4–749.2)	593.2	(499.2–706.1)
1980–1984	718.7	(706.8–731.4)	724.5	(711.6–738.3)	620.2	(575.4–665.6)	597.6	(508.8–689.3)
1985–1989	620.9	(609.5–633.5)	629.4	(616.0–642.8)	543.1	(501.9–583.1)	506.0	(428.7–582.1)
1990–1994	556.7	(545.7–568.1)	574.0	(559.4–587.1)	463.2	(431.8–495.4)	432.6	(378.0–488.7)
1995–1999	495.3	(484.2–506.6)	507.9	(495.0–520.2)	447.1	(417.4–477.5)	415.4	(372.5–462.0)
2000–2004	422.9	(412.6–433.2)	438.1	(425.8–450.7)	406.9	(382.1–431.0)	380.6	(347.0–416.4)
2005–2009	367.8	(357.1–377.6)	401.7	(386.7–416.4)	316.5	(295.8–339.8)	293.2	(260.3–327.0)
Percent change last—first (95% CI)	−57.5%	(−58.8 to −56.2%)	−54.2%	(−56.0 to −52.4%)	−55.8%	(−60.7 to −50.6%)	–52.3%	(−60.8 to −39.7%)
Absolute change last—first (95% CI)	−498.4	(−516.7 to −480.1)	−475.9	(−497.5 to −454.3)	−399.2	(−463.9 to −334.5)	–321.9	(−447.5 to −196.3)
Hungary								
1970–1974	1230.6	(1224.2–1236.0)	1228.5	(1222.5–1234.8)	1244.7	(1214.8–1280.7)	1320.7	(1252.0–1387.9)
1980–1984	1276.5	(1270.2–1282.4)	1274.5	(1268.8–1281.0)	1286.9	(1261.6–1311.4)	1731.8	(1671.0–1797.6)
1990–1994	1213.6	(1207.8–1219.3)	1248.9	(1242.9–1257.8)	1233.5	(1212.2–1257.1)	826.8	(795.2–852.6)
2000–2004	1022.7	(1018.1–1027.1)	1145.8	(1138.9–1153.0)	718.3	(708.4–730.6)	706.1	(683.0–722.3)
Percent change last—first (95% CI)	−16.9%	(−27.5 to −16.4%)	−6.7%	(−7.596 to −6.0%)	−42.3%	(−44.0 to −40.5%)	−46.5%	(−49.5 to −43.4%)
Absolute change last—first (95% CI)	−207.9	(−215.3 to −200.4)	−82.8	(−92.1 to −73.4)	−526.4	(−561.1 to −491.6)	−614.6	(−685.4 to −543.8)

In England and Wales, mortality rates among ‘low’ and ‘middle’ educated were combined because ‘middle’ education is not available in the 1980s and 1990s

*AS MR* age-standardized mortality rate, *NA* not available, *CI* confidence interval

**Fig. 1:**
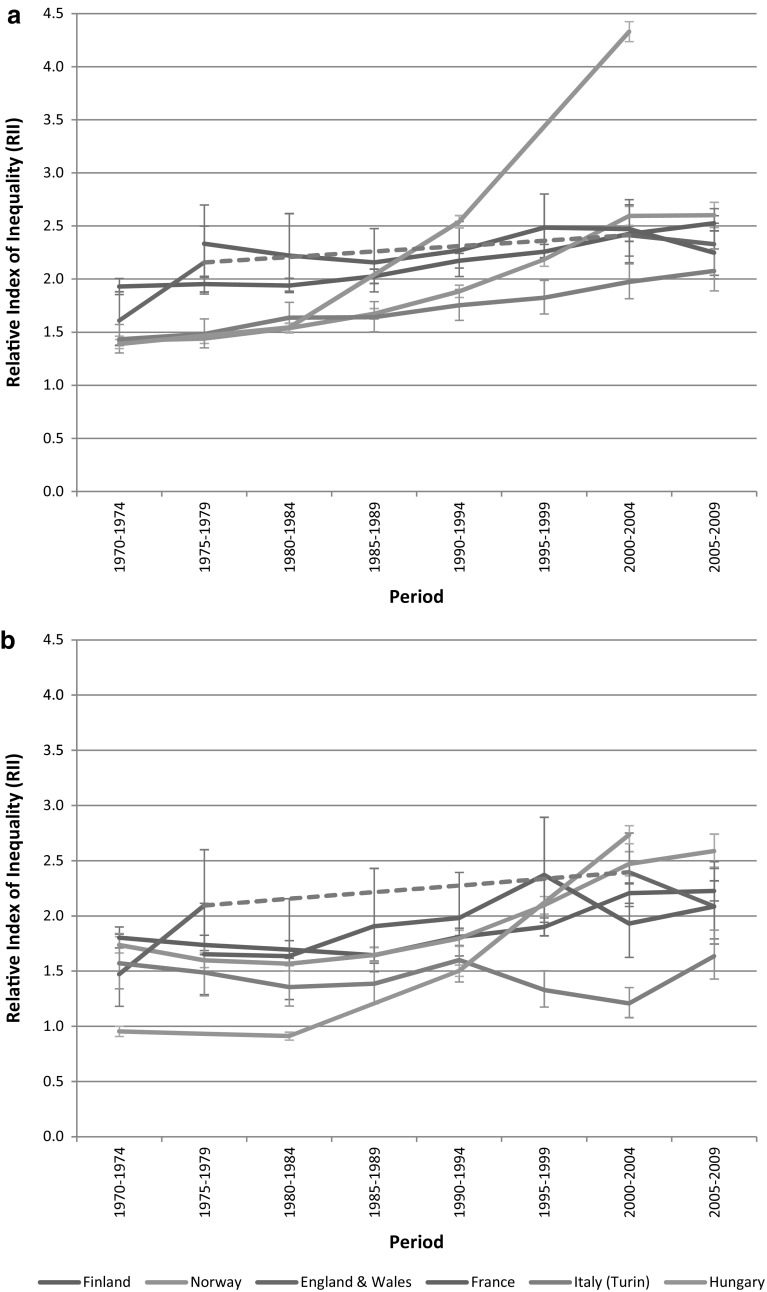
Trends in relative index of inequality for all-cause mortality by education for: **a** men, and **b** women. In England and Wales, RIIs for the period 1980–1999 could not be calculated because ‘middle’ education was not available

Relative (i.e., percentage) declines were almost always largest among the high educated. As a result, relative inequalities in all-cause mortality, as measured by the RII, went up in most countries (Fig. 1). The increase was strongest in Hungary, where the RII went up from a level that was among the lowest among men, and even slightly below 1.00 among women, in the early 1970s to a level that was higher than that in any other country in the early 2000s. Norway also stands out as a country with a relatively steep increase of the RII, both among men and women. In France (men and women) and Italy (women), on the other hand, RIIs did not change significantly over time, as indicated by the overlapping 95% confidence intervals of the first and last observation periods.”

**Fig. 2:**
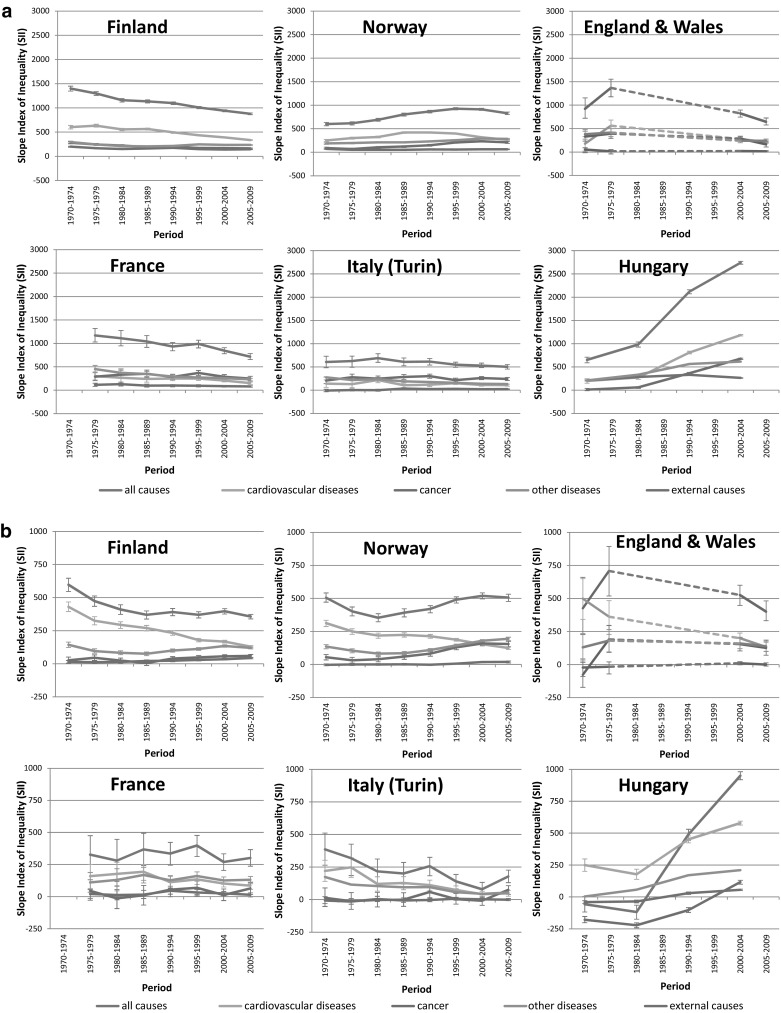
Trends in slope index of inequality for all-cause and cause-specific mortality by education, by country: **a** men, **b** women. In England and Wales, SIIs for the period 1980–1999 could not be calculated because ‘middle’ education was not available

Trends in absolute inequalities in all-cause mortality by education were more variable (Fig. 2). This is due to the fact that absolute declines in mortality were largest among the low educated in some countries [England and Wales, France and Italy (Turin)], but not in others (Norway and Hungary) (Tables 2, 3). Over time, absolute inequalities in mortality as measured by the SII went down among men in most countries, but not in Norway where the SII increased until the late 1990s and only then started to decline (Fig. 2a). In Hungary, due to the enormous rise of mortality among low educated men, the SII for all-cause mortality increased from a relatively low level in the early 1970s to a very high level in the early 2000s.

Among women, although differences in absolute mortality declines between the low and high educated were usually smaller than among men (Tables 2, 3), absolute inequalities in all-cause mortality among women increased in Hungary and Norway, and decreased in Finland, England and Wales and Italy (Turin), as they did in men (Fig. 2b).

Figure 2 also shows that the role of cardiovascular diseases in generating inequalities in all-cause mortality has changed considerably over time, particularly in Finland, England and Wales, and Norway. In these countries, cardiovascular diseases used to be the main contributor to inequalities in all-cause mortality among both men and women, but in the most recent periods this was no longer the case. For example, among Finnish women the contribution of cardiovascular diseases to inequality in all-cause mortality (calculated as 100 × (SII for cardiovascular diseases mortality)/(SII for all-cause mortality)) decreased from 72% in the early 1970s to 36% in the late 2000s. In most countries, absolute decreases in cardiovascular mortality rates were largest among the lower educated (web appendix Table 3), and as a result, absolute inequalities in cardiovascular mortality declined considerably (Fig. 2), although relative inequalities went up (web appendix figure 1).

The declining contribution of cardiovascular diseases to inequality in all-cause mortality implies an increasing contribution of other causes of death: in three out of six countries the contribution of cancer mortality to inequality in all-cause mortality increased among both men and women. The continued increase of absolute inequalities in Norway until the late 1990s was due to a rise of inequalities in cancer and other diseases among both men and women, which more than compensated for the decline of inequalities in cardiovascular disease (Fig. 2). Furthermore, the massive rise of inequalities in all-cause mortality in Hungary was due to rises seen for several causes of death, and to a reversal of formerly ‘negative’ inequalities in cancer mortality (indicating higher mortality among the high educated) to ‘positive’ inequalities in the last observation period.

**Fig. 3:**
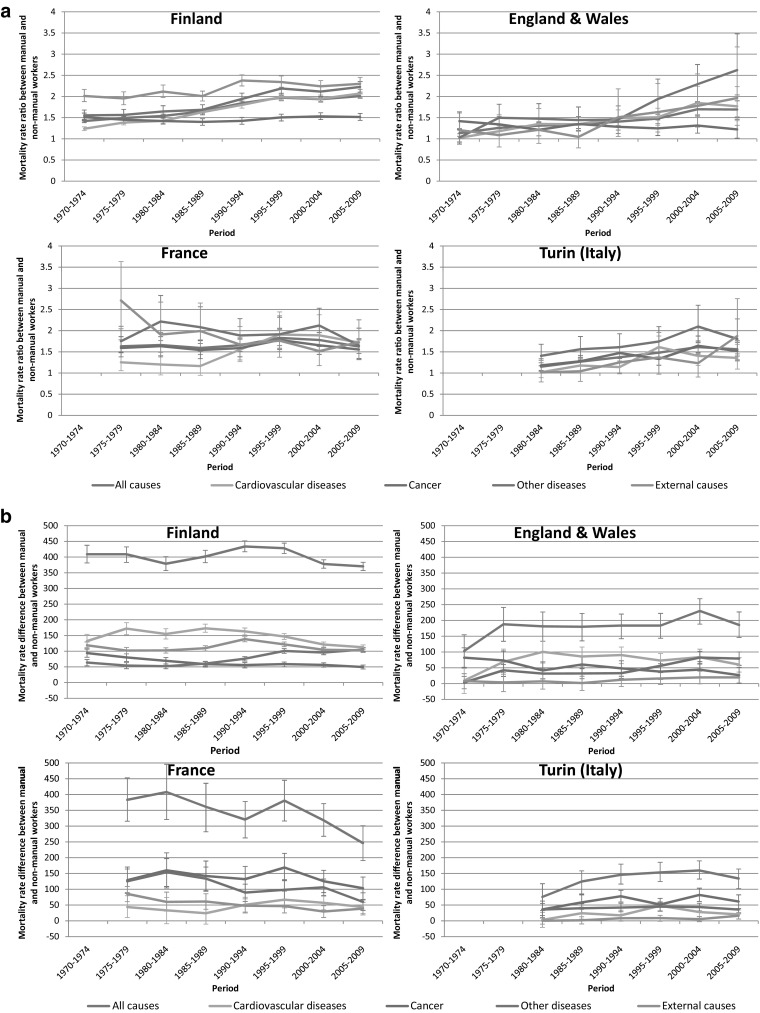
**a** Trends in rate ratio for all-cause and cause-specific mortality by occupational class, by country, men. **b** Trends in rate difference for all-cause and cause-specific mortality by occupational class, by country, men

All-cause mortality trends by occupational class were partly similar to trends by education. In the four countries for which data were available (Finland, England and Wales, France and Italy—men only), mortality rates declined among both manual and non-manual workers, with relative declines usually being largest among non-manual workers, and differences in absolute declines being more variable (web appendix Table 4). This resulted in increasing relative inequalities by occupational class in most countries, as we observed for inequalities by education; however, absolute inequalities decreased in France only, and were stable in the other three countries (Fig. 3).

## Discussion

### Summary

In all countries except Hungary, all-cause mortality declined strongly over time in all socioeconomic groups. Relative inequalities in all-cause mortality generally increased, but much more so in Hungary and Norway than in the other four countries. Absolute inequalities by education often narrowed, particularly among men, but widened in Hungary and Norway. Over time, cardiovascular diseases lost their role as main contributor to inequalities in all-cause mortality in Finland, England and Wales, and Norway. As a result of these trends, Hungary (where inequalities in mortality were almost absent in the 1970s) and Norway (where inequalities in the 1970s among men were amongst the smallest of the six countries in this study) in the most recent period had larger inequalities in mortality than the other four countries.

### Interpretation

It is only over a period of several decades that one can clearly see the very dynamic nature of trends in inequalities in mortality, with some countries that originally had small inequalities in mortality (Hungary and Norway) rising to the top, while others made considerable progress in reducing inequalities in mortality, at least as measured on an absolute scale.

To the best of our knowledge, this is the first study on long-term trends in inequalities in mortality that includes Hungary. Our analysis shows that, contrary perhaps to what might have been expected, the widening of inequalities in mortality already started during the 1980s, i.e., before the fall of the communist regime in 1989 (Kovacs 2014). In the 1970s and 1980s, relative inequalities in mortality in Hungary were among the smallest observed in the countries included in our analysis, but in the early 1990s they were already larger than elsewhere among men, and in the early 2000s were also larger than elsewhere among women (Fig. 1). This explosion of inequalities is due to declining mortality among the high educated at a time when mortality among the low educated was still rising (Tables 2, 3). The early start of these developments suggests that its explanation should not be sought exclusively, or even primarily, in the political and economic changes occurring after the fall of the Soviet Union, although these may have exacerbated the already on-going deterioration of the health status of the low educated (Leinsalu et al. 2009). The fact that cardiovascular disease mortality among the high educated in Hungary already started to decline during the 1980s (web appendix Table 3) suggests that favorable changes in cardiovascular risk factors (e.g., dietary changes, or smoking cessation) and/or treatment of cardiovascular disease had by then already reached the high educated in Hungary (Kovacs 2014). Figure 2b shows that the ‘reverse’ inequalities in all-cause mortality seen among Hungarian women in the early 1970s and early 1980s were primarily due to higher cancer mortality among the high educated. Cancers for which mortality was higher among the high educated include those of breast and lung (results not shown), suggesting that ‘modern’ child-bearing and smoking behaviors had already been adopted by high but not by low educated women in Hungary in these years (Strand et al. 2007).

It is perhaps no surprise that Hungary, with its different political history, appears to inhabit a different world in terms of health inequalities, but there is also a stark contrast between Norway and the other Western- and Southern European countries. Relative inequalities in Norway have steeply risen, and absolute inequalities have increased as well, at least until the first half of the 2000s. This is partly related to a rise in inequalities in mortality due to cardiovascular diseases until the late 1980s among men, and a more recent rise in inequalities in mortality due to cancer and other diseases among both men and women (Fig. 2). Inequalities in mortality from lung cancer have risen substantially in Norway among both men and women, while they are already declining among men in other Northern and Western European countries, suggesting that a delayed smoking epidemic partly explains Norway’s unfavorable trajectory (Gregoraci et al. 2016, accepted for publication). The fact that inequalities in cardiovascular disease are already declining in Norway, whereas inequalities in lung cancer are still increasing, despite the fact that both diseases are related to smoking, can probably be explained by differences in the lag-time between smoking and the occurrence of these two diseases.

Relative inequalities among French men and women and Italian women also stood out because they remained stable, partly due to strong declines in mortality from other diseases and external causes among the low educated. Excessive alcohol consumption plays a role in mortality from both other diseases (e.g. in the form of liver cirrhosis) and external causes, and as we have shown elsewhere, while inequalities in alcohol-related mortality have increased in many countries in Northern, Western and Central/Eastern Europe, they have not in Southern Europe, possibly because of between-country variations in alcohol control policies and in changes in the affordability of alcohol (Mackenbach et al. 2015).

The secular change of declining rates of cardiovascular disease mortality has been hailed as opening a new stage in the epidemiologic transition (Olshansky and Ault 1986), but also as one of the factors underlying the widening of inequalities in mortality in recent decades (Marmot and McDowall 1986). Widening of inequalities in mortality has even been proposed as an indicator of the start of a new stage in the epidemiologic transition (Vallin and Meslé 2004). However, our results show that whether or not one sees a widening occurring depends strongly on the perspective chosen. Absolute inequalities in cardiovascular disease mortality have often declined, because mortality decline was stronger in absolute terms among the low than among the high educated. Declines in mortality from ischemic heart disease and cerebrovascular disease over the past decades in high-income countries have been attributed to a combination of risk factor changes (e.g. changes in smoking, blood pressure, cholesterol, physical activity) and improvements in treatment of these conditions (e.g., revascularization, secondary prevention) (Hunink et al. 1997; McGovern et al. 1992; Sarti et al. 2000; Unal et al. 2004). Larger absolute declines in mortality from cardiovascular disease among the low than the high educated indicate that these developments have also considerably benefitted the low educated, as has indeed been demonstrated empirically for England (Bajekal et al. 2012; Scholes et al. 2012). This suggests that reach of prevention programs and access to treatment among lower socioeconomic groups are crucial for reducing inequalities in mortality. Nevertheless, our data also show that relative inequalities in cardiovascular mortality have increased rapidly, due to larger percentage reductions in cardiovascular mortality among the high than the low educated. Therefore, inequalities in cardiovascular disease mortality remain a reason for concern, although they now no longer stand out as the most important contributor to inequalities in all-cause mortality in Northern and Western Europe.

### Strengths and limitations

This is the first study of long-term trends in socioeconomic inequalities in mortality covering 4 decades and 6 European countries. Such a broad coverage naturally also comes with certain limitations. For example, in Italy only data from the city of Turin could be included. However, recent national-level studies from Italy (Federico et al. 2013; Marinacci et al. 2013) found inequalities that were similar to those that were observed in this study, which suggests that Turin does not misrepresent the whole country.

Questions may also be raised on the between- and within-country comparability of the data. For instance, variations between and within countries in the certification and coding of causes of death may have biased our results. However, this should not be a substantial problem in our study, because misclassification is most likely to happen within, and not between, the broad categories of causes of death analyzed here. Potentially more important are differences and changes in the classification of educational level. Most countries provided longitudinal linked data, in which educational information for both the numerator and denominator of mortality rates came from the census, but Hungary had unlinked cross-sectional data only. In countries with cross-sectional data numerator/denominator bias can occur, due to the fact that relatives of a deceased sometimes give incorrect information on the educational level of that person. As a consequence, educational differences in mortality could be under- or overestimated in Hungary (Shkolnikov et al. 2012). However, the type of data did not change over time, and therefore the within-country trend analysis, which was the main aim of our study, is unlikely to be biased.

Educational systems and classifications may also have changed over time. We reclassified national educational levels into the ISCED scheme (UNESCO 2006), and then re-categorized these categories into 3 broader educational levels, which should have removed most differences in classification within and between countries. However, categorizing education into ‘low’, ‘middle’ and ‘high’ level entailed some difficulties. In England and Wales, for instance, ‘middle’ level education had been grouped with ‘low’ education during the 1980s and 1990s. For England and Wales, RIIs and SIIs could only be calculated for the 1970s and 2000s (for which 3 education groups were originally available), and inequality trends between these periods were estimated based on RIIs/SIIs in 1975–1979 and 2000–2004. As a sensitivity analysis, inequalities were also measured as mortality rate ratio and rate difference between those with low and high education for all time periods, which yielded similar results (web appendix figure 2a and b).

Furthermore, in Hungary, vocational education was categorized as ‘low’ instead of ‘middle’ level education (which is recommended by ISCED guidelines), because it was part of compulsory education before the Hungarian educational system was reformed in the 1970s. As a consequence, we likely have underestimated inequalities in Hungary during the whole study period to some extent.

Both education and occupational class were included as indicators of socioeconomic position. Even though results were to some extent similar, discrepancies between the two measures were also observed: in Finland, England and Wales and Italy (Turin) absolute inequalities by education went down, whereas those by occupational class were largely stable or increased. These discrepancies may be due to problems in classification, particularly for occupational class (e.g., due to the exclusion of economically inactive), or to differences in the age group that was analyzed (35–79 for education, 35–64 for occupation), but there may also be substantive explanations, e.g., in terms of differences between the two indicators in capturing various aspects of socioeconomic position. Unfortunately, no data on mortality by occupational class were available for Norway and Hungary, the two countries with the most unfavorable trends in educational inequalities.

Our analysis covered the age group 35–79 years at death (35–64 for analyses by occupation), and thereby excludes younger age groups, and also under-represents older age groups which currently carry the largest share of the burden of mortality, particularly in Western Europe where average life expectancy at birth in the 2000s exceeded 75 years for men and 80 years for women. This implies that our results cannot necessarily be generalized to mortality in the whole population. The slightly older population in Norway (aged 40–79 at death) may have resulted in somewhat higher absolute inequalities, but are unlikely to have affected the observed trends of inequalities.

### Conclusions

There is no agreement among researchers or policy-makers on whether to use relative or absolute measures for monitoring progress towards reduction of health inequalities (Mackenbach and Kunst 1997; Harper et al. 2010). We tend to think that while both are important, in the end absolute inequalities matter most, because ultimately it is the absolute excess death rate in lower socioeconomic groups that affects people’s lives, not the relative excess of a more and more infrequent event (Mackenbach 2015). It is therefore very good news that absolute inequalities are narrowing over time in several European countries.

Over this 40-year period, trends in inequalities in mortality have been very dynamic, with large variations between countries. This shows that inequalities in mortality are not immutable, and that while some countries have experienced dramatic setbacks, others have made substantial progress in reducing inequalities in mortality. The diminishing role of cardiovascular disease in generating inequalities in all-cause mortality further suggests that policies to reduce inequalities in mortality should start to focus more on other diseases, including cancer and alcohol-related conditions.

## Electronic supplementary material

Below is the link to the electronic supplementary material.

Supplementary material 1 (DOCX 463 kb)
