# Supplementary material for: Long-term trends of inequalities in mortality in 6 European countries
Source: Int J Public Health. 2016 Dec 9;62(1):127–41. doi: 10.1007/s00038-016-0922-9 (PMC5288439; doi:10.1007/s00038-016-0922-9)
Supplement: Supplementary file 1 — Supplementary material 1 (DOCX 463 kb) [file 38_2016_922_MOESM1_ESM.docx]

**LONG-TERM TRENDS OF INEQUALITIES IN MORTALITY IN 6 EUROPEAN COUNTRIES**

**Web appendix**

**Long-term trends of inequalities in mortality in six European countries.**

International Journal of Public Health.

De Gelder R, Menvielle G, Kovacs K, Martikainen P, Strand BH, Mackenbach JP.

Corresponding author: Prof. Dr. Johan P. Mackenbach. Department of Public Health, Erasmus MC, Erasmus University Medical Center, Rotterdam, The Netherlands. E-mail: j.mackenbach@erasmusmc.nl

**Web appendix figure 1a. Trends in Relative Index of Inequality for all-cause and cause-specific mortality by education, men**

**
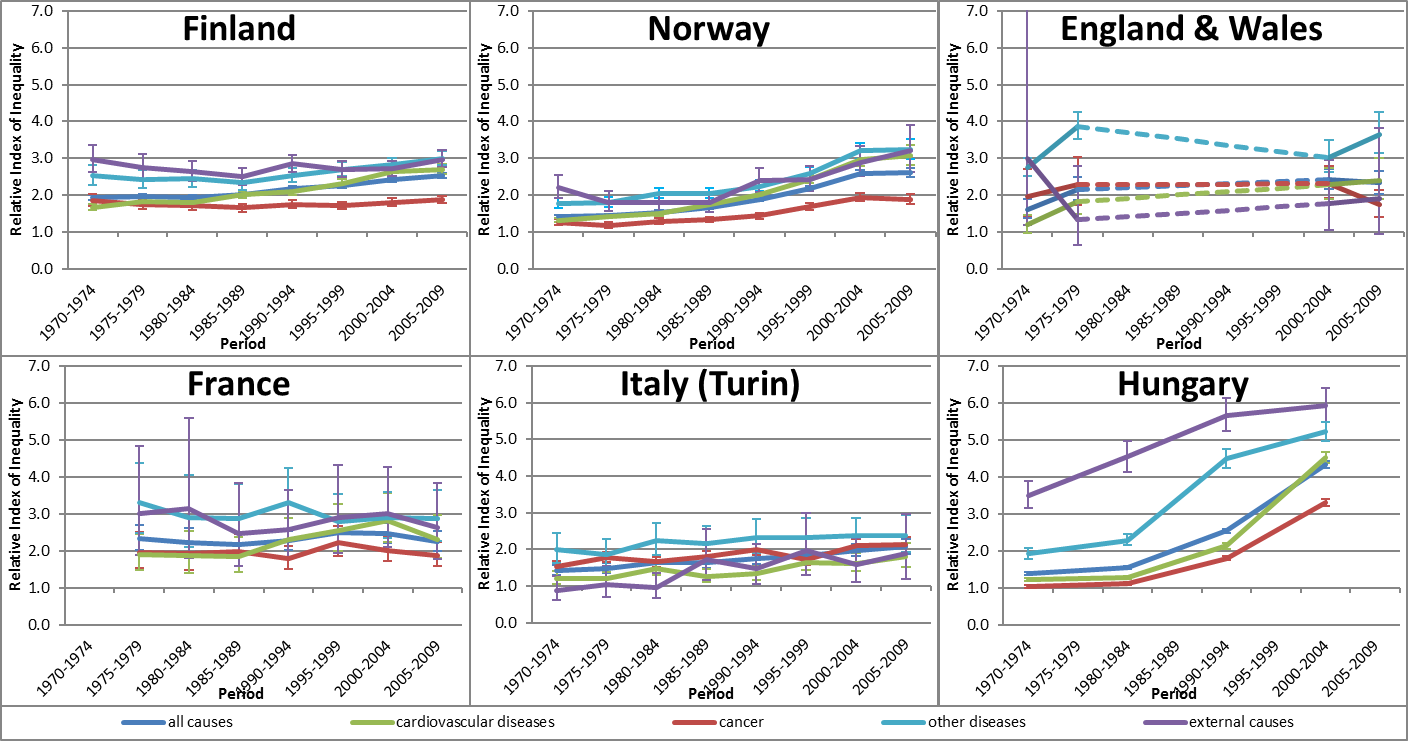
**

In England & Wales, RIIs for the period 1980-1999 could not be calculated because ‘middle’ education was not available.

A trend line was estimated based on the RIIs in 1975-1979 and 2000-2004, and is indicated with a dashed line.

**Web appendix figure 1b. Trends in Relative Index of Inequality for all-cause and cause-specific mortality by education, women**

**
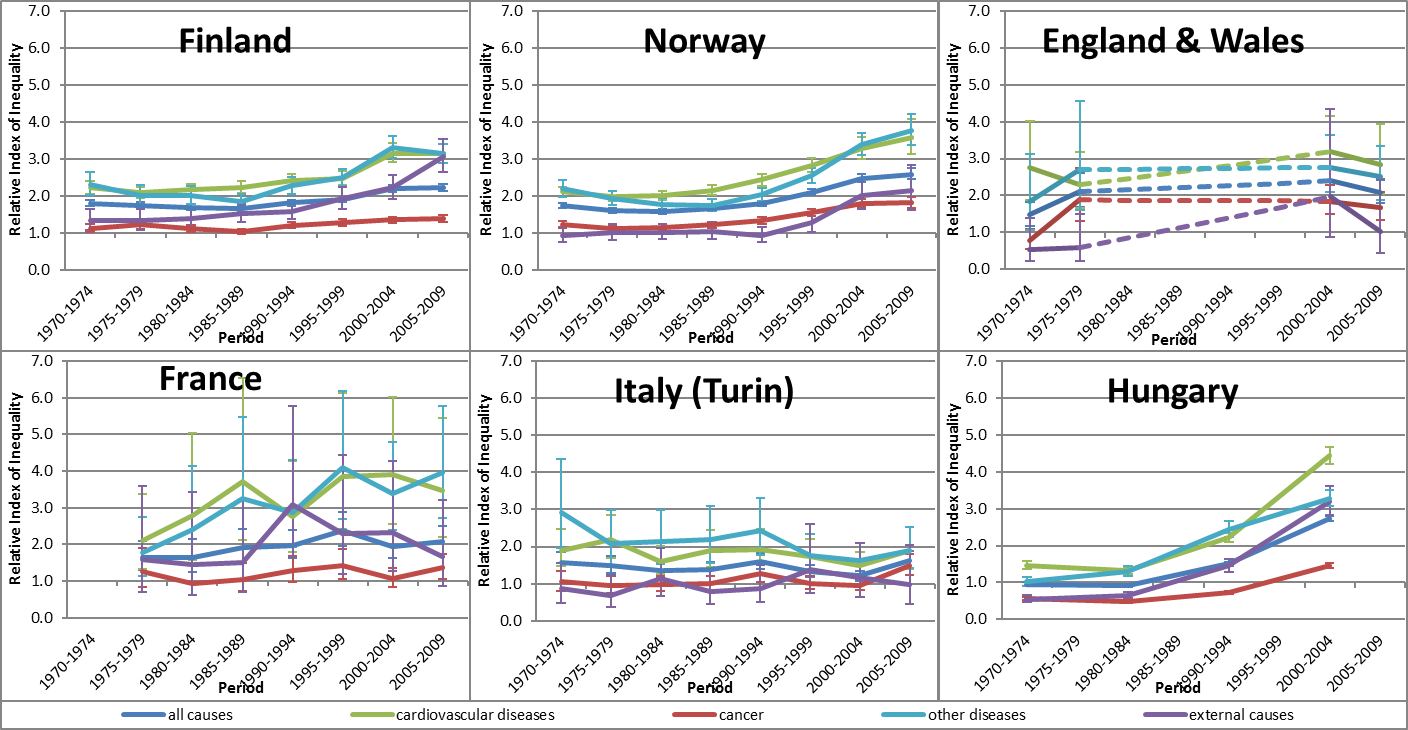
**

In England & Wales, RIIs for the period 1980-1999 could not be calculated because ‘middle’ education was not available.

A trend line was estimated based on the RIIs in 1975-1979 and 2000-2004, and is indicated with a dashed line.

**Web appendix figure 2. Trends in rate difference (a) and rate ratio (b) for all-cause mortality**

**a.**

**
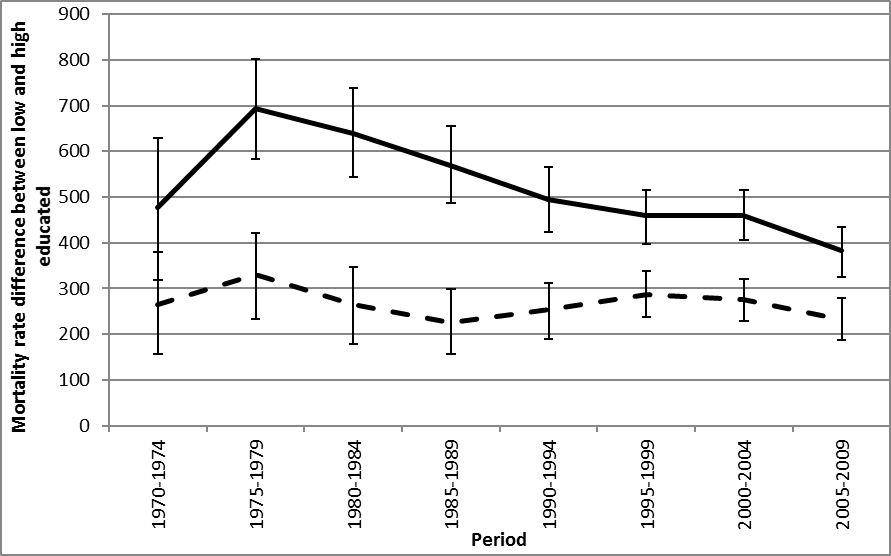
**

**b.**

**
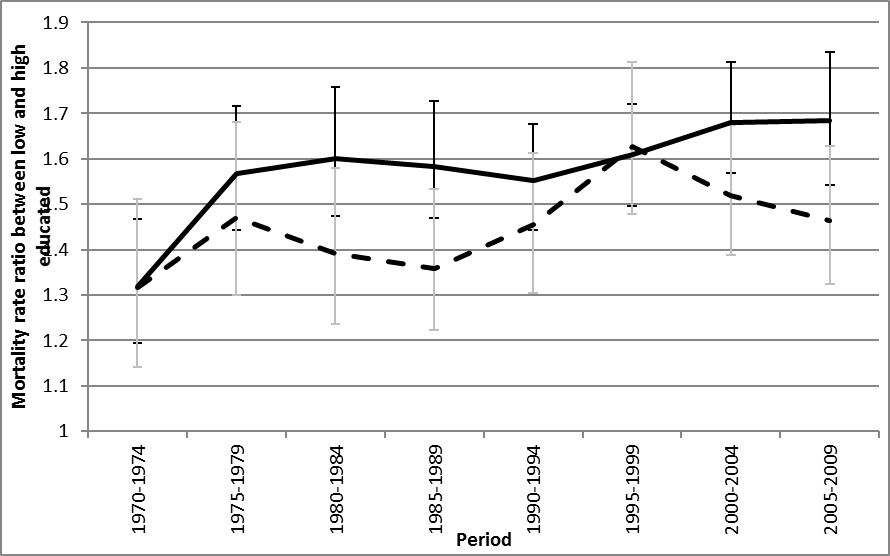
**

**
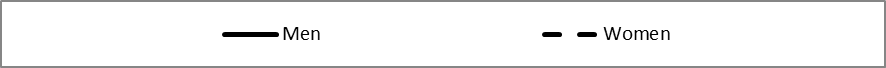
**

**Web appendix table 1. Educational distribution of the populations included in the analysis**

| Country | Census date | Missing | ISCED 0-2 (%) | ISCED 3-4 (%) | ISCED 5-6 (%) |
| --- | --- | --- | --- | --- | --- |
| Finland | 31.12.1970 | - | 79.8 | 11.3 | 8.9 |
|  | 31.12.1980 | - | 67.9 | 18.2 | 13.9 |
|  | 31.12.1990 | - | 52.5 | 27.6 | 19.9 |
|  | 31.12.2000 | - | 38.1 | 34.6 | 27.3 |
| Norway | Nov, 1970 | 2.9 | 62.2 | 31.5 | 6.3 |
|  | Nov, 1980 | 3.2 | 54.5 | 36.7 | 8.8 |
|  | Nov, 1990 | 3.3 | 42.3 | 43.3 | 14.4 |
|  | Nov, 2001 | 3.3 | 31.3 | 46.7 | 22 |
| England/ Wales | 25.4. 1971 | 7.1 | 90.3 | 3.2 | 6.6 |
|  | 5.4.1981 | 0 | 91.1 | - | 8.9 |
|  | 21.4.1991 | 7 | 84.8 | - | 15.2 |
|  | 29.4. 2001 | 9.3 | 59.7 | 20.6 | 19.6 |
| France | 10.1.1975 | - | 78.3 | 16.6 | 5.2 |
|  | 10.1.1982 | - | 70.5 | 21.7 | 7.7 |
|  | 10.1.1990 | - | 57.9 | 31.1 | 11.1 |
|  | 10.1.1999 | - | 43.2 | 39.9 | 16.9 |
| Italy (Turin) | 25.10.1981 | 1.8 | 84.3 | 11 | 4.7 |
|  | 20.10.1991 | - | 75.6 | 16.7 | 7.7 |
|  | 21.10.2001 | 0 | 63.6 | 24.4 | 11.9 |
| Hungary | 1.1.1973 | - | 88.1 | 7.9 | 4 |
|  | 1.1.1980 | - | 83.1 | 11.4 | 5.5 |
|  | 1990 | - | 74 | 16.4 | 9.6 |
|  | 2001 | - | 64.3 | 23.3 | 12.4 |

ISCED: International Standard Classification of Education 1997

**Web appendix table 2. ICD codes**

|  | **ICD 8 codes** | **ICD 9 codes** | **ICD 10 codes** |
| --- | --- | --- | --- |
| Cardiovascular diseases | 390-458 | 390-459 | I00-I99 |
| Cancer | 140-239 | 140-239 | C00-D48 |
| Other diseases | Rest (001-E999) | Rest (001-E999) | Rest (A00-U85) |
| External causes | E800-E999 | E800-E999 | V01-Y98 |

ICD: International Classification of Diseases

**Web appendix table 3a: Cause-specific mortality rates and changes between first and last observation period, by educational group, men**

ASMR: Age-standardized mortality rate

In England and Wales, mortality rates among ‘ low’ and ‘ middle’ educated were combined because ‘middle’ education is not available in the 1980s and 1990s

**Web appendix table 3b: Cause-specific mortality rates and changes between first and last observation period, by educational group, women**

ASMR: Age-standardized mortality rate

In England and Wales, mortality rates among ‘ low’ and ‘ middle’ educated were combined because ‘middle’ education is not available in the 1980s and 1990s

**Web appendix table 4. All-cause and cause-specific mortality rates and changes between first and last observation period, by occupational class, men**

ASMR: Age-standardized mortality rate
